# Arthroscopic popliteus bypass graft for posterolateral instabilities of the knee: A new surgical technique

**DOI:** 10.1007/s00064-015-0432-6

**Published:** 2015-12-04

**Authors:** K.-H. Frosch, R. Akoto, T. Drenck, M. Heitmann, C. Pahl, A. Preiss

**Affiliations:** Department of Trauma and Reconstructive Surgery, Asklepios Clinic St. Georg, Lohmühlenstr. 5, 20099 Hamburg, Germany; Division of Knee and Shoulder Surgery, Sports Traumatology, Asklepios Clinic St. Georg, Hamburg, Germany

**Keywords:** Knee, Posterior cruciate ligament, Posterolateral instability, Popliteus tendon, Posterolateral corner, Knie, Hinteres Kreuzband, Posterolaterale Instabilität, Popliteussehne, Posterolaterale Ecke

## Abstract

**Objective:**

An arthroscopic technique for the reconstruction of the posterolateral corner combined with posterior cruciate ligament (PCL) reconstruction was developed.

**Indications:**

Posterolateral rotational instabilities of the knee. Combined lesions of the PCL, the popliteus complex (PLT) and the posterolateral corner. Isolated PLT lesions lacking static stabilizing function.

**Contraindications:**

Neuromuscular disorders; knee deformities or fractures; severe posterolateral soft tissue damage.

**Surgical technique:**

Six arthroscopic portals are necessary. Using the posteromedial portal, resect dorsal septum with a shaver. Visualize the PCL, the lateral femoral condyle and the posterolateral recessus with the PLT. Dissect the popliteomeniscal fibers; retract PLT until sulcus popliteus is visualized. Drill a 6-mm tunnel anteriorly into the distal third of the sulcus popliteus. Visualize femoral footprint of the PLT and place an anatomical drill tunnel. Pull the popliteus bypass graft into the knee and fix with bioscrews. Fix the reconstructed PCL. In cases of additional LCL injury, reconstruct LCL with autologous graft.

**Postoperative management:**

Partial weight-bearing for 6 weeks, range of motion exercises, quadriceps-strengthening exercises on postoperative day 1. Full extension allowed immediately with flexion limited to 20° for 2 weeks, to 45° for up to week 4, and to 60° up to week 6. Use a PCL brace for 3 months, running and squatting exercises allowed after 3 months.

**Results:**

In the 35 patients treated, no technique-related complications. After 1 year, 12 patients had a mean Lysholm Score of 88.6 (± 8.7) points and a side-to-side difference in the posterior drawer test of 2.9 (± 2.2) mm (preoperative 13.3 [± 1.9] mm).

**Conclusion:**

Low complication risk and good and excellent clinical results after arthroscopic posterolateral corner reconstruction.

## Zusammenfassung

**Operationsziel:**

Entwicklung einer neuen arthroskopischen Technik zur kombinierten Rekonstruktion der posterolateralen Ecke und des hinteren Kreuzbands (HKB).

**Indikationen:**

Posterolaterale Rotationsinstabilitäten des Kniegelenks. Kombinierte Läsionen des HKB, des Popliteuskomplexes (PLT) und der posterolateralen Ecke. Isolierte PLT-Läsionen mit posterolateraler Instabilität.

**Kontraindikationen:**

Neuromuskuläre Störungen, Kniedeformitäten und -frakturen, schwere Weichteilschäden in der posterolateralen Region.

**Operationstechnik:**

Notwendig sind 6 arthroskopische Portale. Über das posteromediale Portal wird das dorsale Septum reseziert und HKB sowie posterolateraler Rezessus mit dem PLT dargestellt. Am Hiatus popliteus werden popliteomeniskale Fasern durchtrennt, die Popliteussehne aus dem Sulcus luxiert und der distale Anteil des Sulcus popliteus dargestellt. Bohren eines 6-mm-Bohrkanals von ventral in das distale Drittel des Sulcus popliteus. Der femorale Popliteussehnenansatz wird dargestellt und ein anatomischer femoraler Bohrkanal gesetzt. Einziehen des Grazilissehnentransplantats als „Popliteus Bypass Graft“ und Fixation mit Biotenodeseschrauben. Fixation des HKB-Transplantats. Bei einer LCL-Instabilität zusätzliche Rekonstruktion des LCL.

**Postoperative Behandlung:**

Bodenkontaktbelastung für 6 Wochen, Orthese mit Bewegungslimitierung für 3 Monate, Quadrizepskräftigungsübungen direkt postoperativ. Sofort volle Streckung, sukzessive Beugungsfreigabe bis 90° über 6 Wochen. Joggen oder tiefe Kniebeugen frühestens nach 3 Monaten.

**Ergebnisse:**

Keine technikspezifischen Komplikationen bei 35 behandelten Patienten. Nach 1 Jahr hatten 12 Patienten einen mittleren Lysholm-Score von 88,6 (± 8,7) Punkten und eine radiologisch gemessene dorsale Instabilität im Seitenvergleich von 2,9 (± 2,2) mm (präoperativ 13,3 [± 1,9] mm).

**Schlussfolgerung:**

Niedrige Komplikationsraten und gute erste klinische Ergebnisse nach arthroskopischer Rekonstruktion der posterolateralen Ecke.

## Introductory remarks

**Figure 1:**
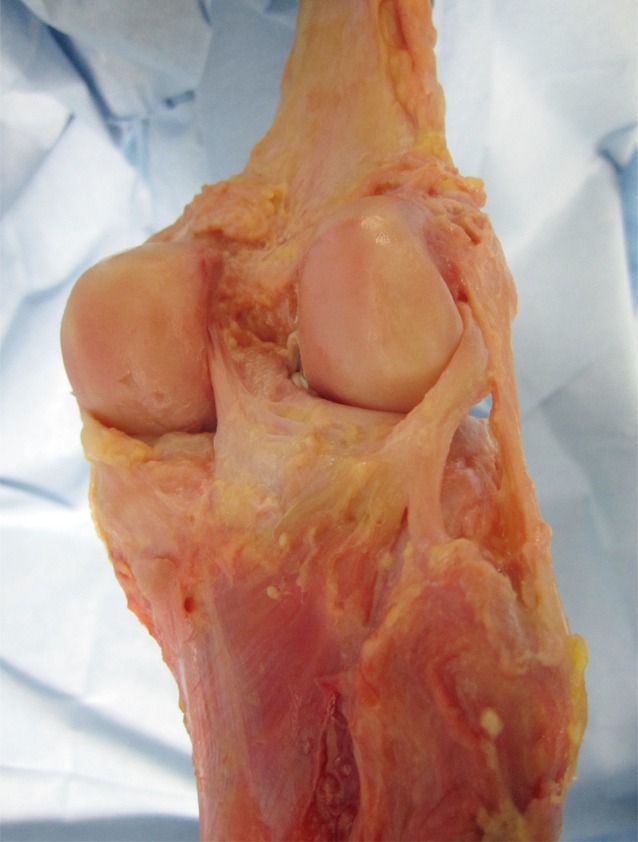

**Figure 2:**
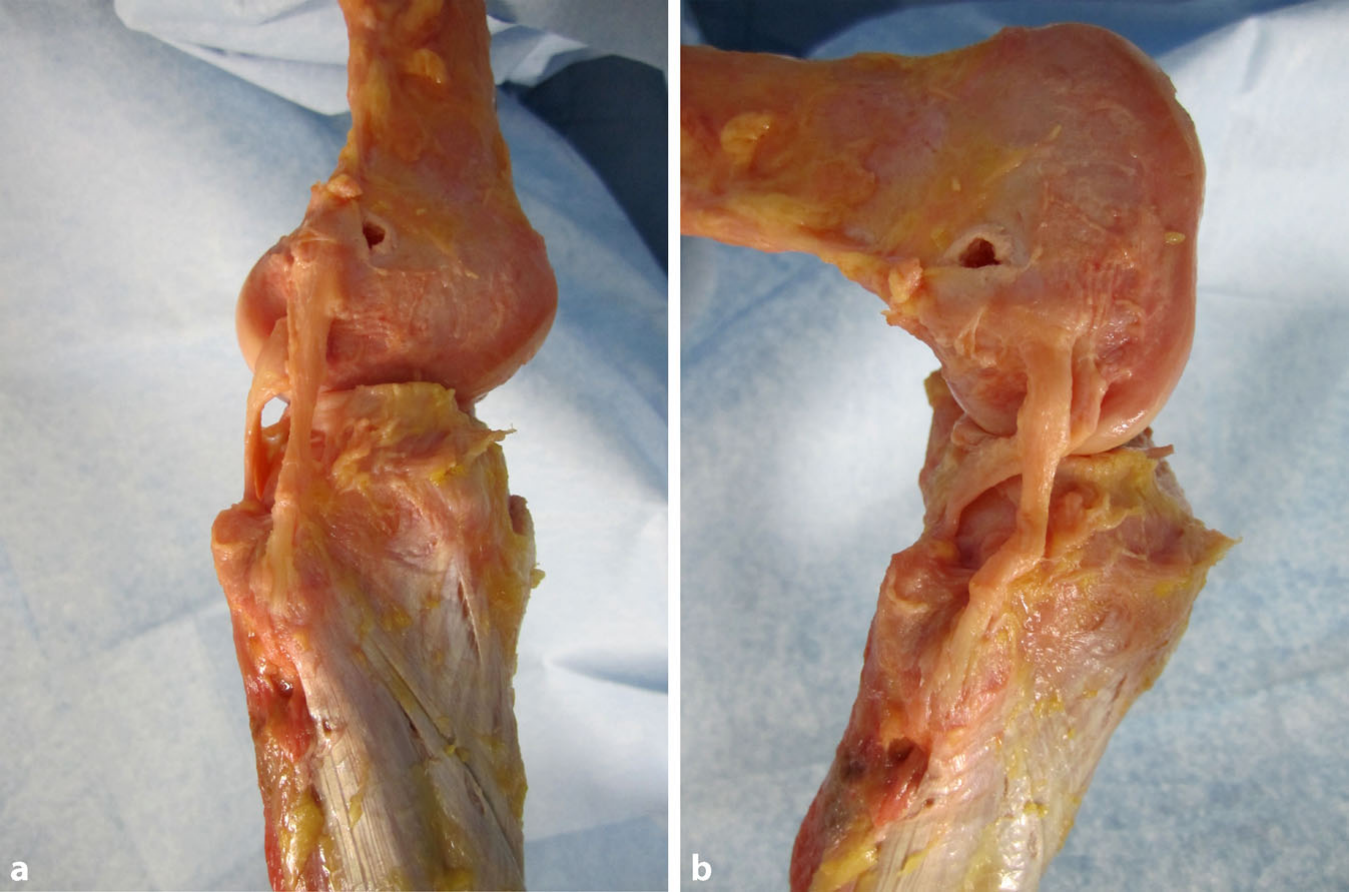

The anatomy of the knee is complex, and particularly that of the posterolateral corner ([8]; Fig. 1). The popliteus tendon complex has a static and a dynamic function. The popliteus muscle–tendon (PLT) itself acts in a dynamic function as an active internal rotator of the tibia and adjusts the postural equilibrium during standing [14]. The static biomechanical function of resistance against passive external rotation of the tibia is achieved in combination with the arcuate complex (AC; [11]). The AC mainly comprises the popliteofibular ligament, the fabellofibular ligament, popliteomeniscal fibers, and multiple extensions of the popliteus tendon to the tibia and to the posterior capsule (Fig. 1). Thereby, the AC represents the primary static stabilizer to external rotation [10, 15, 21]. The most prominent structure of the AC is the popliteofibular ligament (Fig. 1, 2a, b).

The great importance of the AC for stabilization of the tibia against external rotation especially in flexion has been described previously [10, 15, 17]. If the AC is injured, primary posterior translation and coupled external rotation [13, 21] increases. With an isolated injury of the posterior cruciate ligament (PCL), a posterior instability of up to 10 mm in 90° of flexion results [16]. Additional dissection of the PLT results in a dorsal instability of up to 15 mm in the posterior drawer test in 90° of flexion. These biomechanical results indicate that a dorsal instability of more than 10 mm in 90° of flexion results in a combined posterolateral rotational instability [16]. Up to 70 % of all PCL injuries are combined injuries with additional lesions of the posterolateral corner [8, 16].

For an exact analysis of the kind of instability (dorsal, lateral, rotational, posterolateral, or combined), it has to be considered that the main constraint against tibial external rotation from 0 to 30° is the LCL, while the arcuate complex becomes dominant towards increasing flexion, exhibiting its main function at 90° of flexion [3, 5, 9]. In addition, the LCL is the main stabilizer against varus stress in 0–30° extension. In this study, patients with a posterolateral rotational instability including a posterior drawer of more than 10 mm underwent reconstruction of the popliteus complex with a popliteus bypass graft in combination with PCL reconstruction. LCL was additionally reconstructed only if a lateral instability in 10° of flexion was evident.

The first anatomical reconstruction of the popliteus complex with an anatomical popliteus bypass graft was described by Werner Müller in 1982 [12]. Thereafter, numerous surgical techniques to reconstruct the static stabilizing function of the PLT have been described [1, 4, 6, 7, 18, 20, 22, 23]. Most of these are extraanatomical techniques with limited capacity to stabilize the posterolateral corner. With anatomical techniques for the reconstruction of the posterolateral corner, good and excellent results have been described [9, 19]. However, the described techniques are basically open surgical procedures, without the advantages of an arthroscopic technique.

We therefore developed a novel arthroscopic procedure for anatomic reconstruction of the popliteus complex with a popliteus bypass graft [2]. In this paper, the operative technique is presented in detail.

## Surgical principles and objectives

The goal of the surgical procedure is to regain the static stabilizing function of the popliteus complex. The dynamic stabilizing function of the popliteus complex should thereby be preserved. These goals should be achieved by an arthroscopic procedure with exact and anatomic tunnel placement [2].

## Advantages

- Restoration of the anatomy and biomechanics of the knee by anatomical reconstruction
- Proper visualization of anatomical landmarks, which is not possible with open techniques
- Utilization of small incisions with a greater likelihood of lower infection rates, lower rates of scar tissue formation, less postoperative pain, faster rehabilitation, and more aesthetic incisions
- Preparation and visualization of the peroneal nerve are not necessary

## Disadvantages

- Requires advanced arthroscopic skills
- Requires experience in PCL and PLT surgery due to the demanding technique
- The use of special instruments is strongly recommended (i. e., tibial drill guide)
- A flat learning curve

## Indications

- Posterolateral rotational instabilities of the knee joint

## Contraindications

- Fixed dorsal position of the tibia (i. e., after ACL reconstruction)
- Systemic diseases like rheumatoid arthritis, autoimmune diseases, etc.
- Neuromuscular disorders
- Anatomic deformities and acute fractures around the knee
- Obesity (relative)

## Patient information

- General risk factors related to arthroscopic surgery: infection, complex regional pain syndrome, deep vein thrombosis, pulmonary embolism, neurovascular iatrogenic injuries, failure
- Duration of hospital stay: 3–4 days
- Persistent instability
- Arthrofibrosis with limited range of motion
- Possible development of degenerative joint disease over time
- Graft harvesting from the contralateral side
- Possibility of iatrogenic damage to the infrapatellar branch of the saphenous nerve or the peroneal nerve
- Prolonged rehabilitation protocol: full extension is allowed immediately, flexion is limited, brace for 3 months (i. e., Jack PCL, Albrecht, Munich, Germany)
- Clinical assessment at 3, 6, 9, and 12 months
- Surgical failure may require another open procedure
- Running and squatting exercises are allowed after 3 months
- High-level sports may commence 6–9 months after surgery
- Return to work/sports activities are dependent on the type of work/sports

## Preoperative and diagnostic work-up

**Figure 3:**
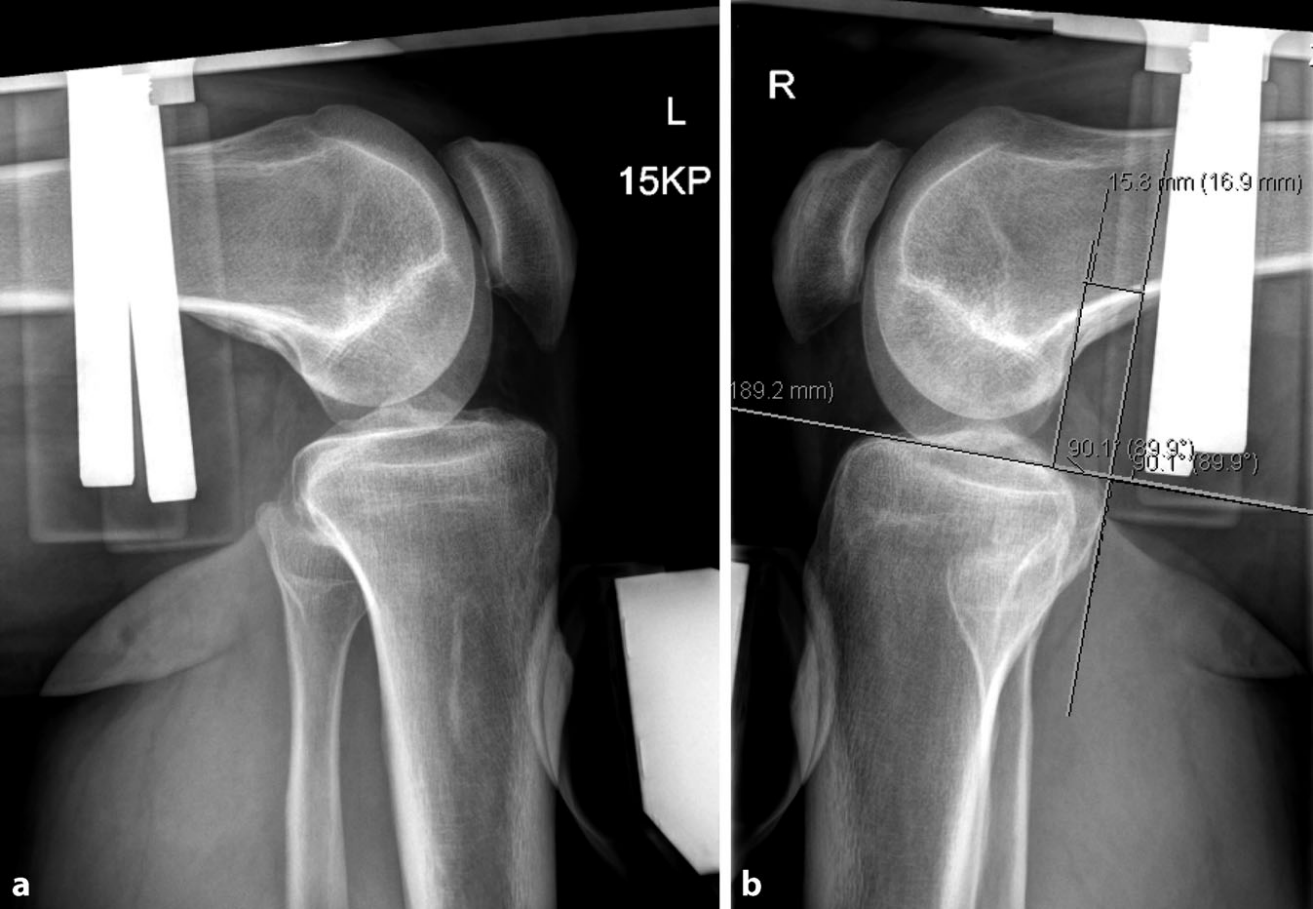

- Patient history
- Clinical assessment with posterior drawer and Dial tests
- Anterior and medial instability should be ruled out
- Lateral stability test in full extension (LCL) and 10° and 90° of flexion
- External rotation test at 30, 60, and 90° in comparison to the contralateral side
- Brace test (optional, to test whether patient’s symptoms improve by using a PCL brace)
- Fixed dorsal drawer should be ruled out
- MRI assessment of the knee
- Anterior–posterior, lateral, and long x-ray views (in clinically suspected cases of axis deviation)
- Stress x-rays with anterior and posterior drawer of both knees (Fig. 3)
- Preoperative management to assure good range of motion (> 0–0–100°)
- Intensive quadriceps strengthening preoperatively
- Side which is planned for operation should be marked prior to surgery

## Surgical instruments and implants

- Arthroscopic instruments: hook, grasper, shaver (4 mm blade, not too sharp), radiofrequency electrode, guide wires, tendon harvester, drill bits in different sizes (6–10 mm), WORM (Arthrex, Naples, FL, USA)
- Drill guide for PCL reconstruction and a special drill guide for arthroscopic posterolateral corner reconstruction (Tibial Popliteal Marking Hook, Arthrex, Naples, FL, USA)
- Biointerference screws for graft fixation in different diameters (5–9 mm) (Milagro, DePuy Mitek, Norderstedt, Germany or Swivelock, Arthrex, Naples, FL, USA, etc.)

## Anesthesia and positioning

**Figure 4:**
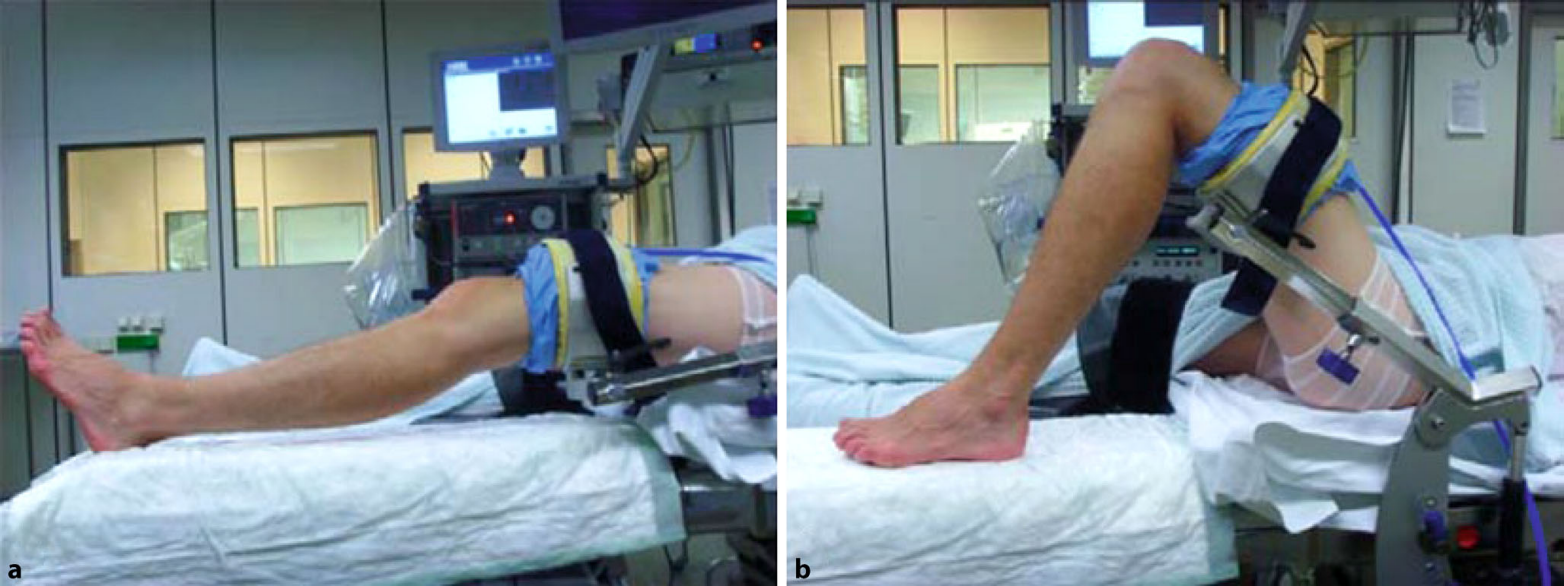

- General or spinal anesthesia
- Supine position
- Non-sterile thigh tourniquet
- Electrical leg holder (Maquet, Germany; Fig. 4)

## Surgical technique

(Figs. 5, 6, 7, 8, 9, 10, 11, 12, 13, 14, 15, 16)

**Figure 5:**
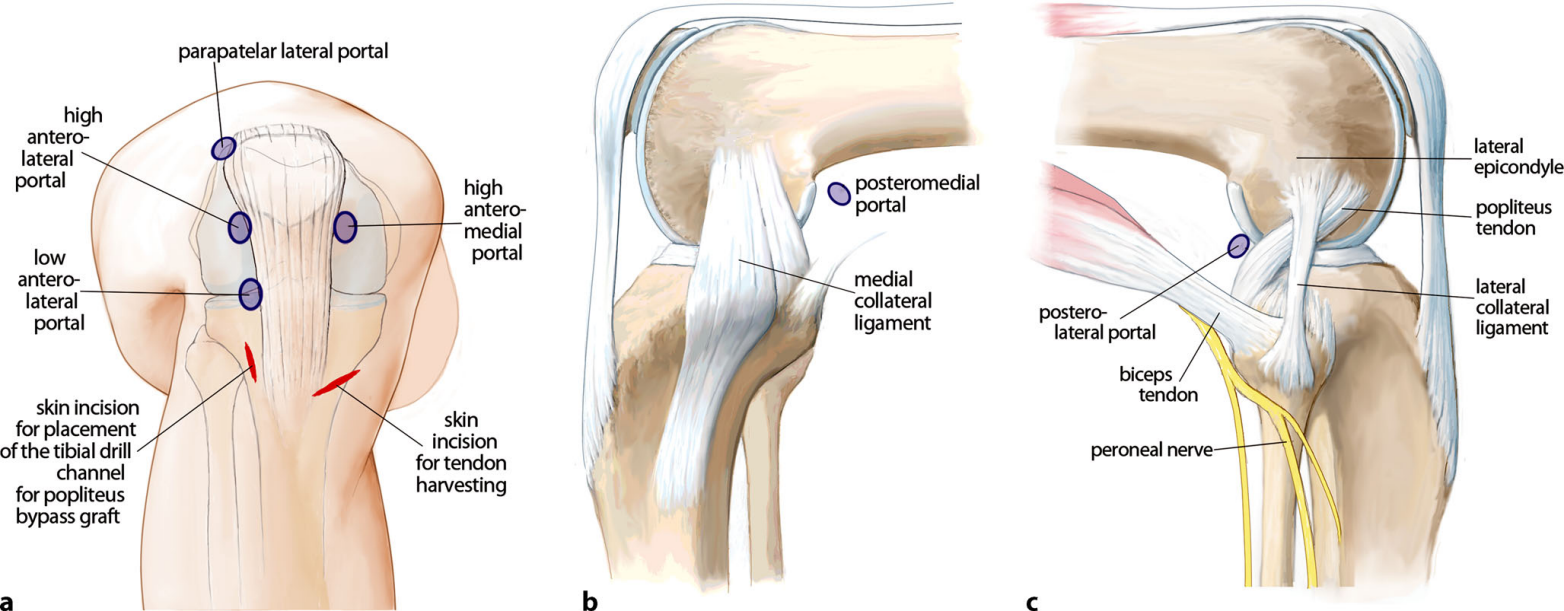

**Figure 6:**
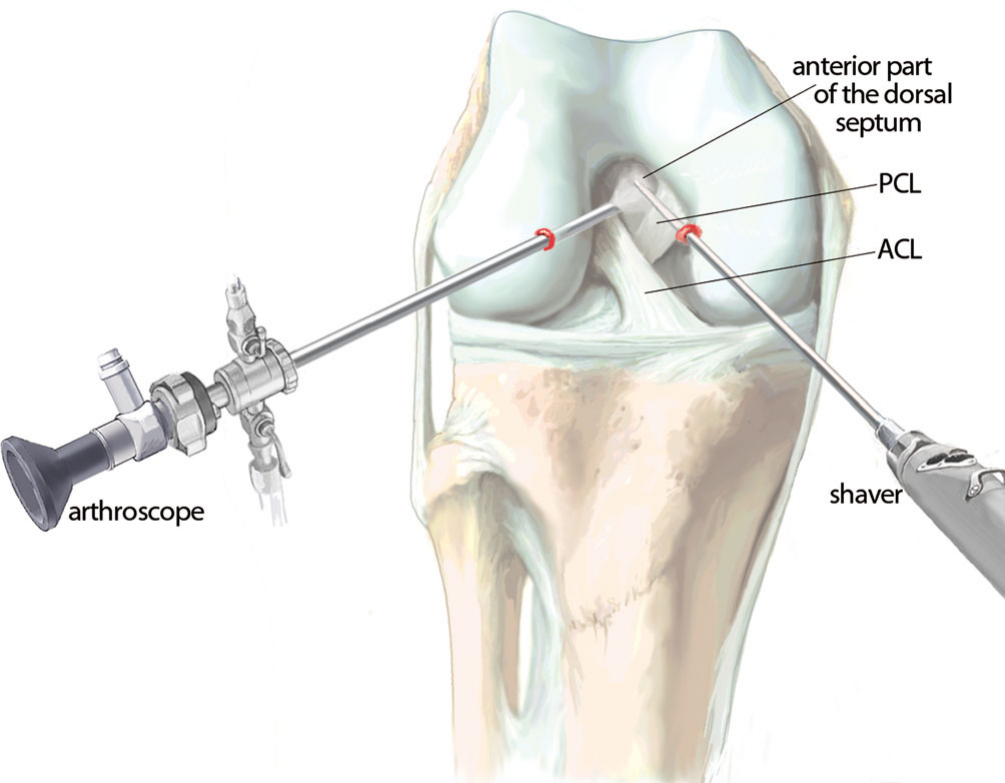

**Figure 7:**
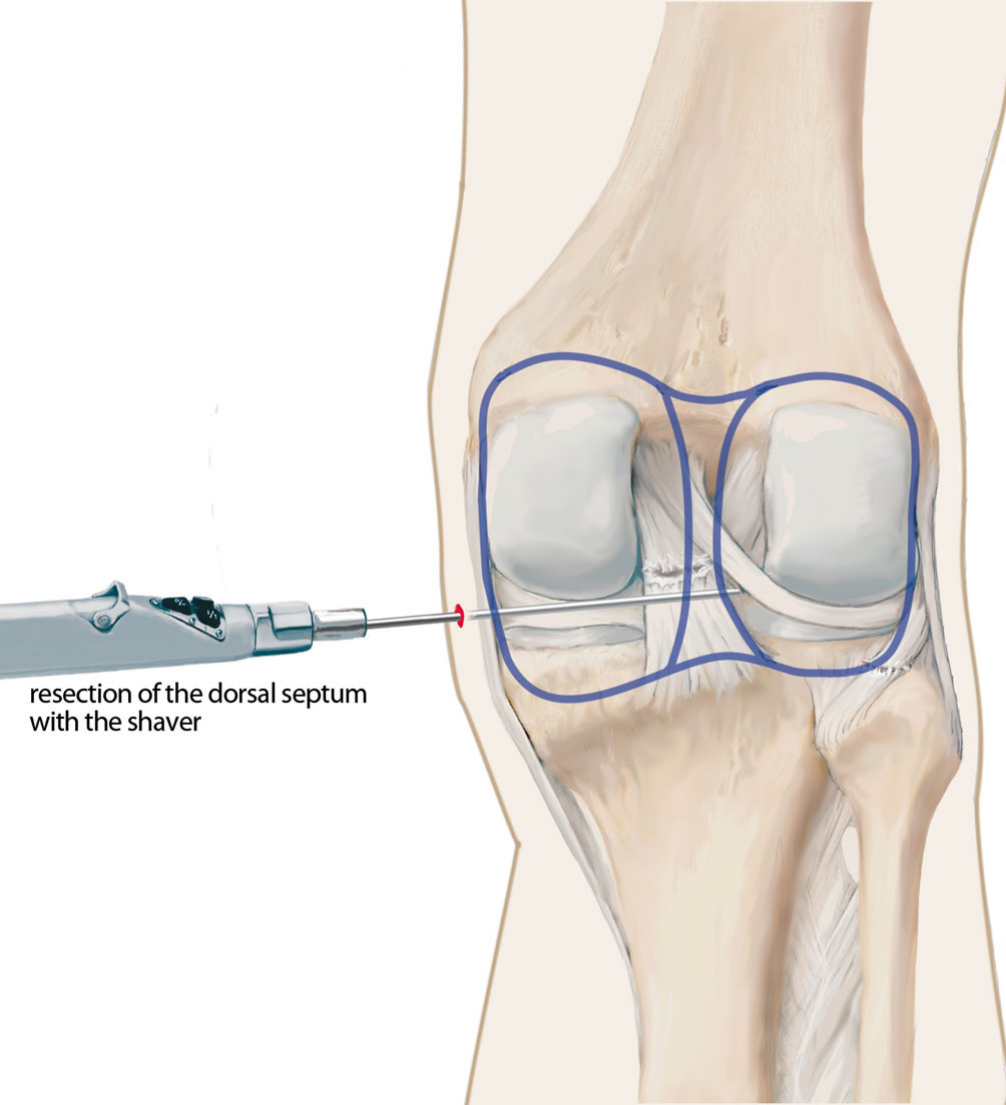

**Figure 8:**
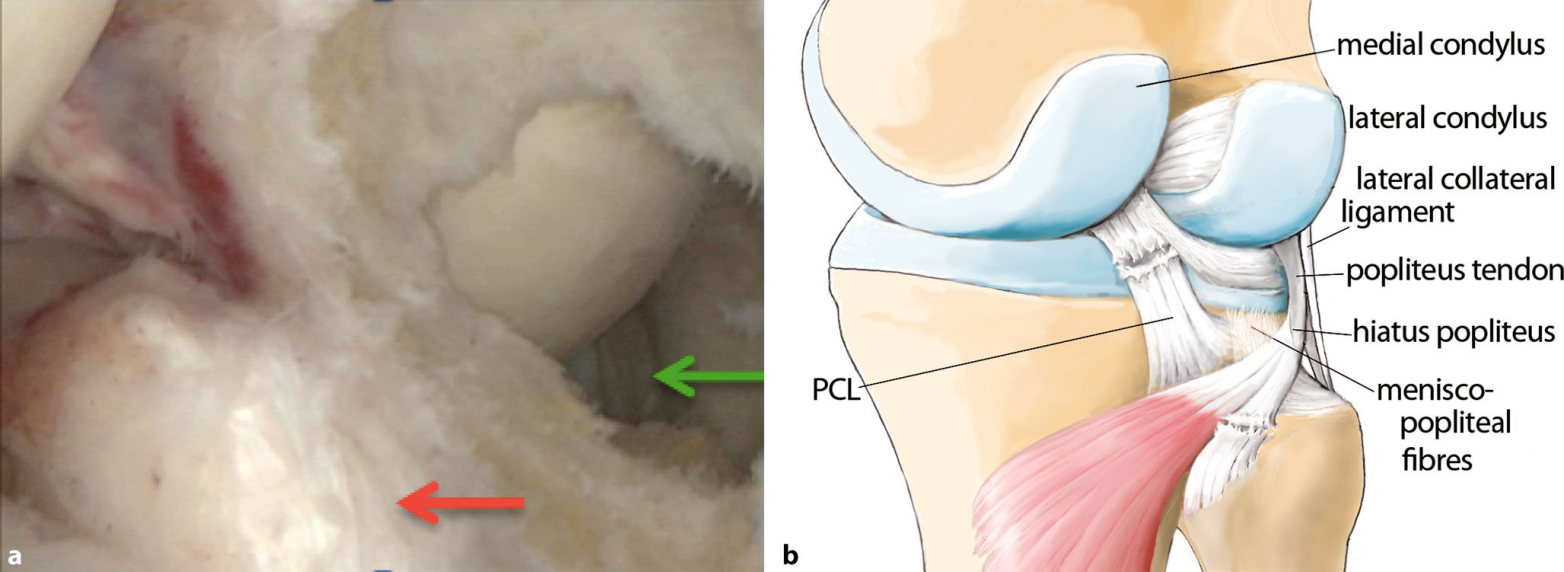

**Figure 9:**
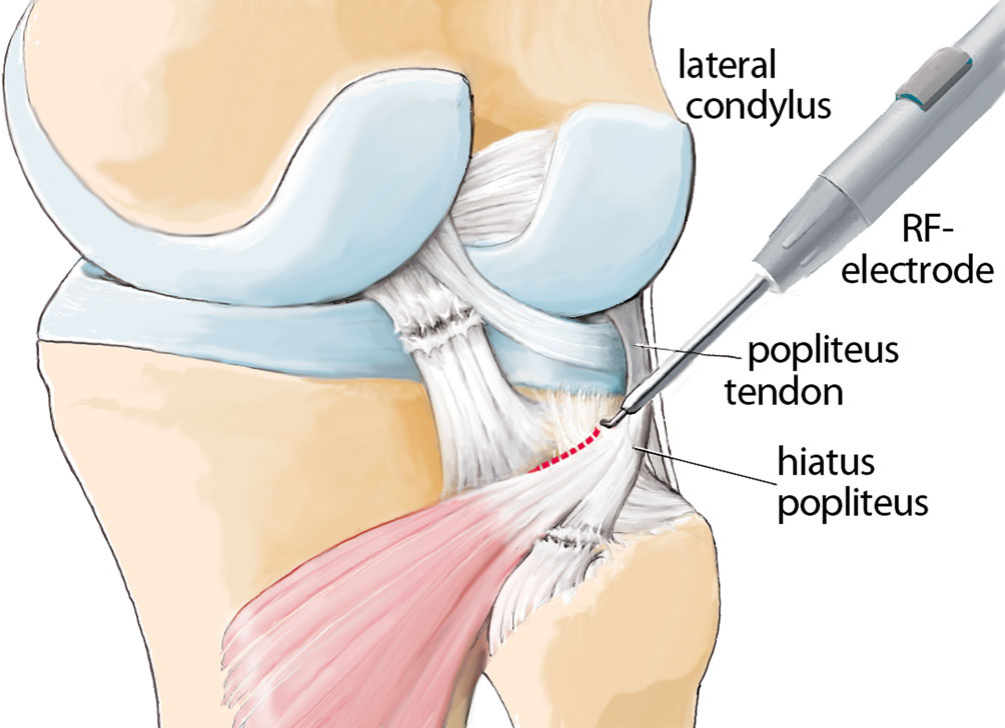

**Figure 10:**
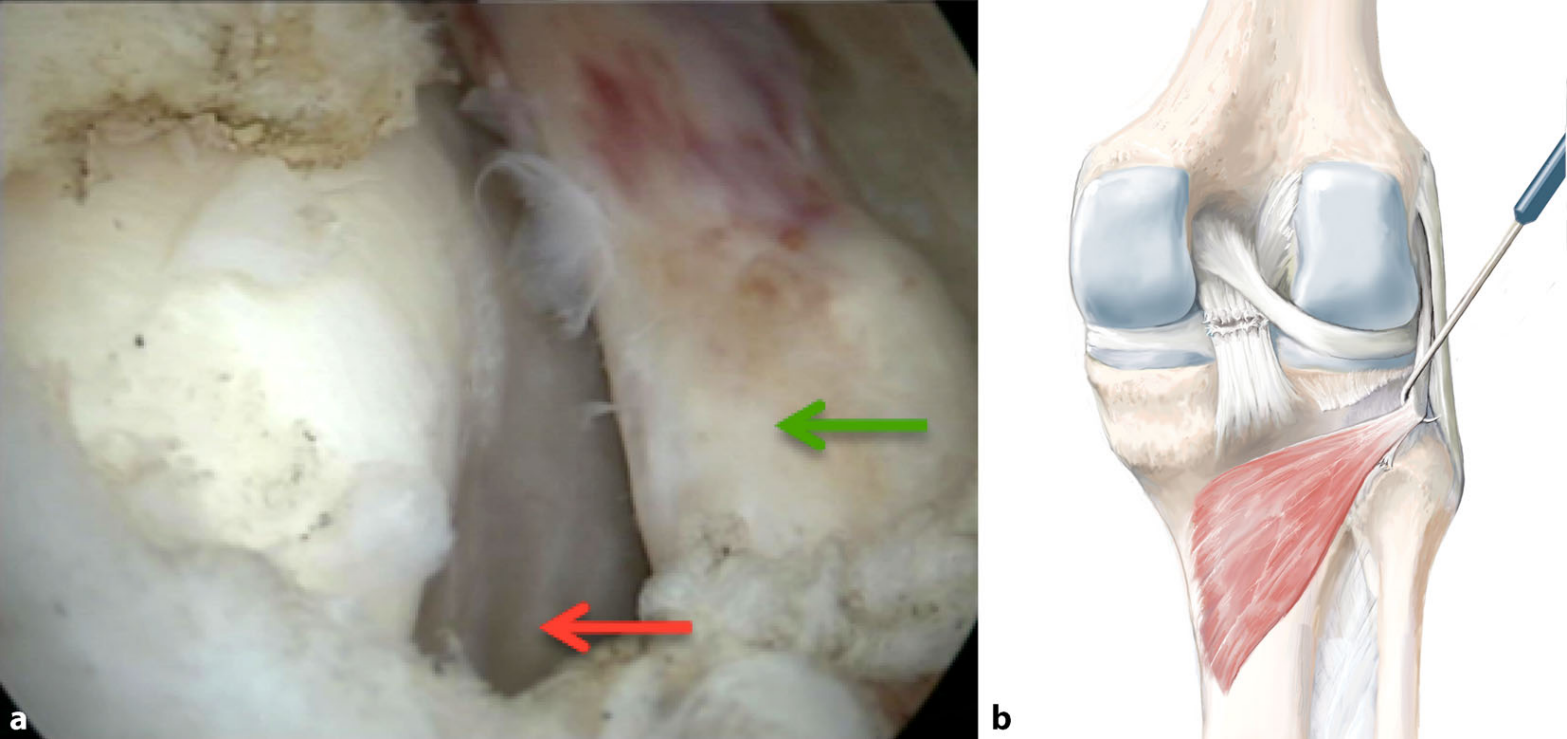

**Figure 11:**
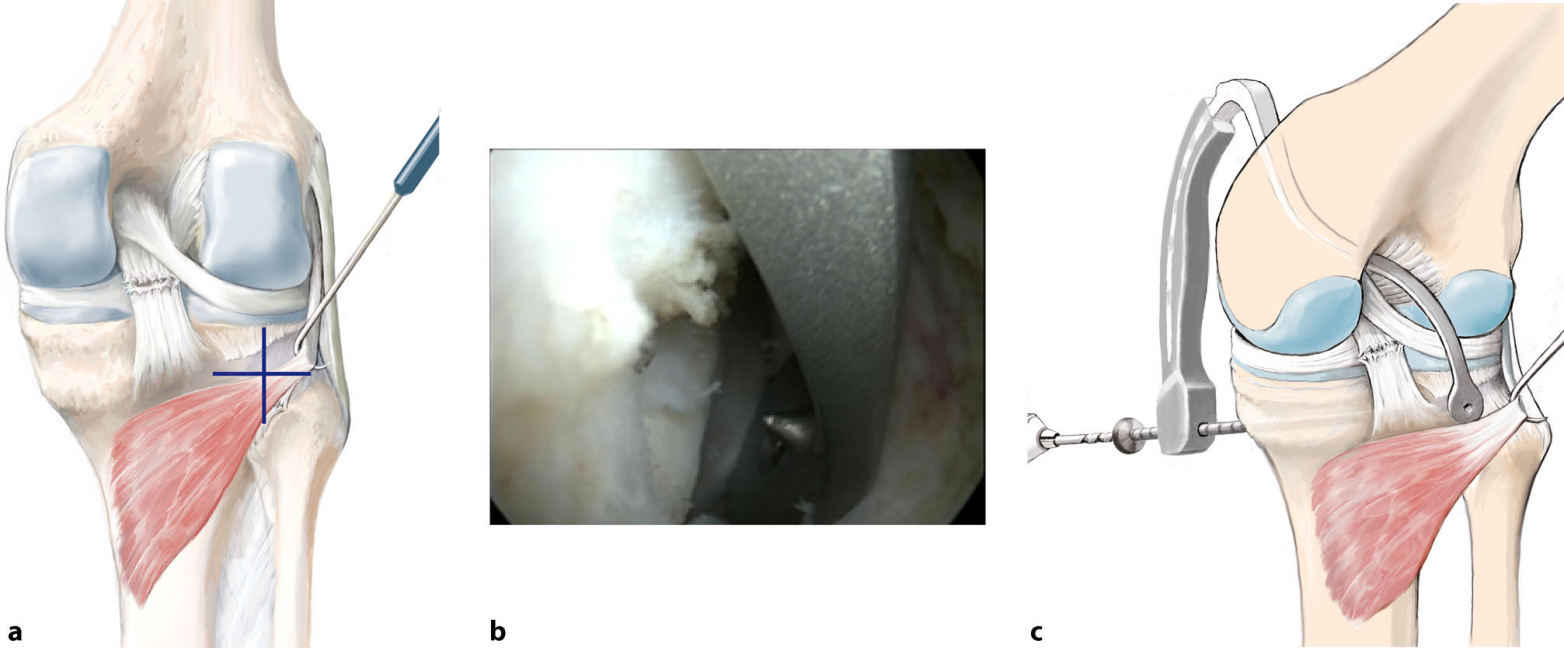

**Figure 12:**
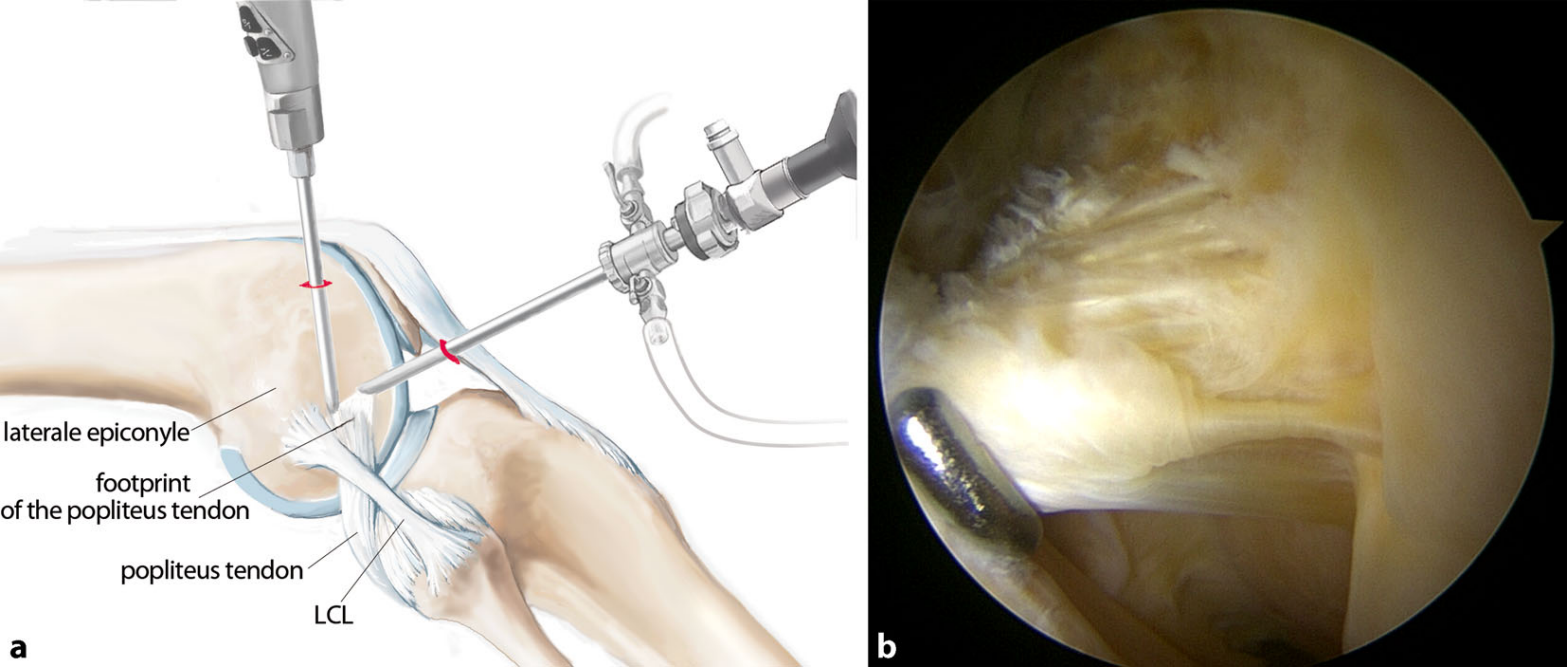

**Figure 13:**
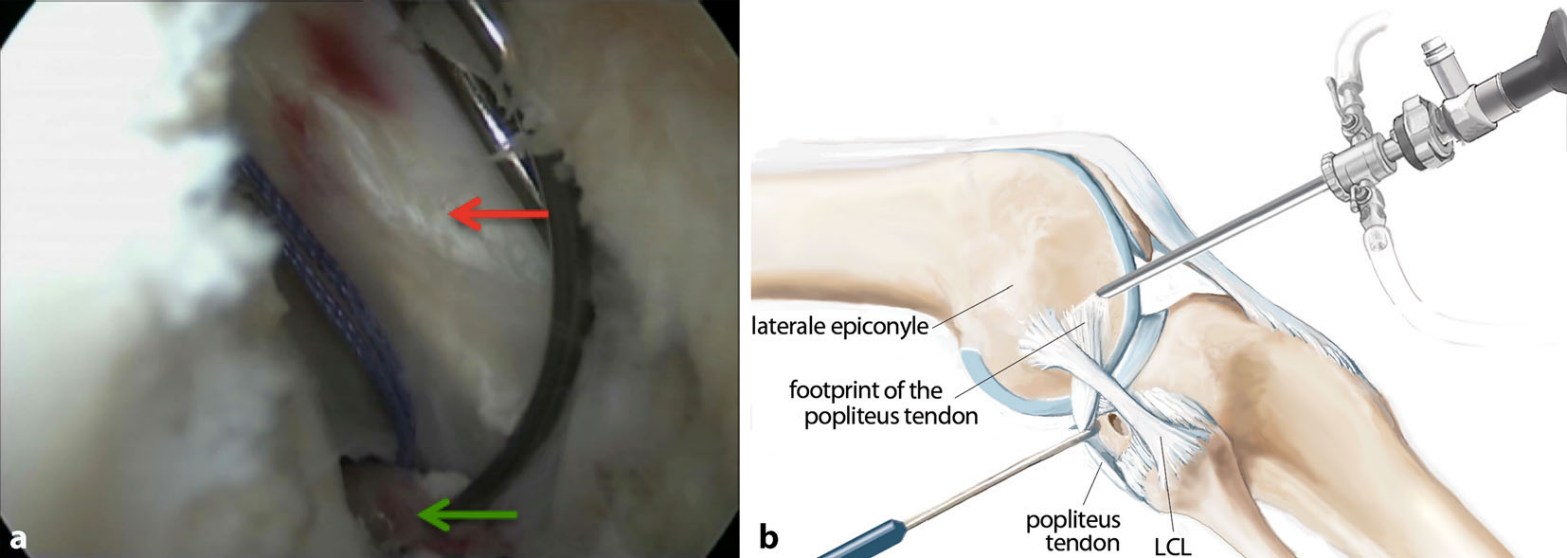

**Figure 14:**
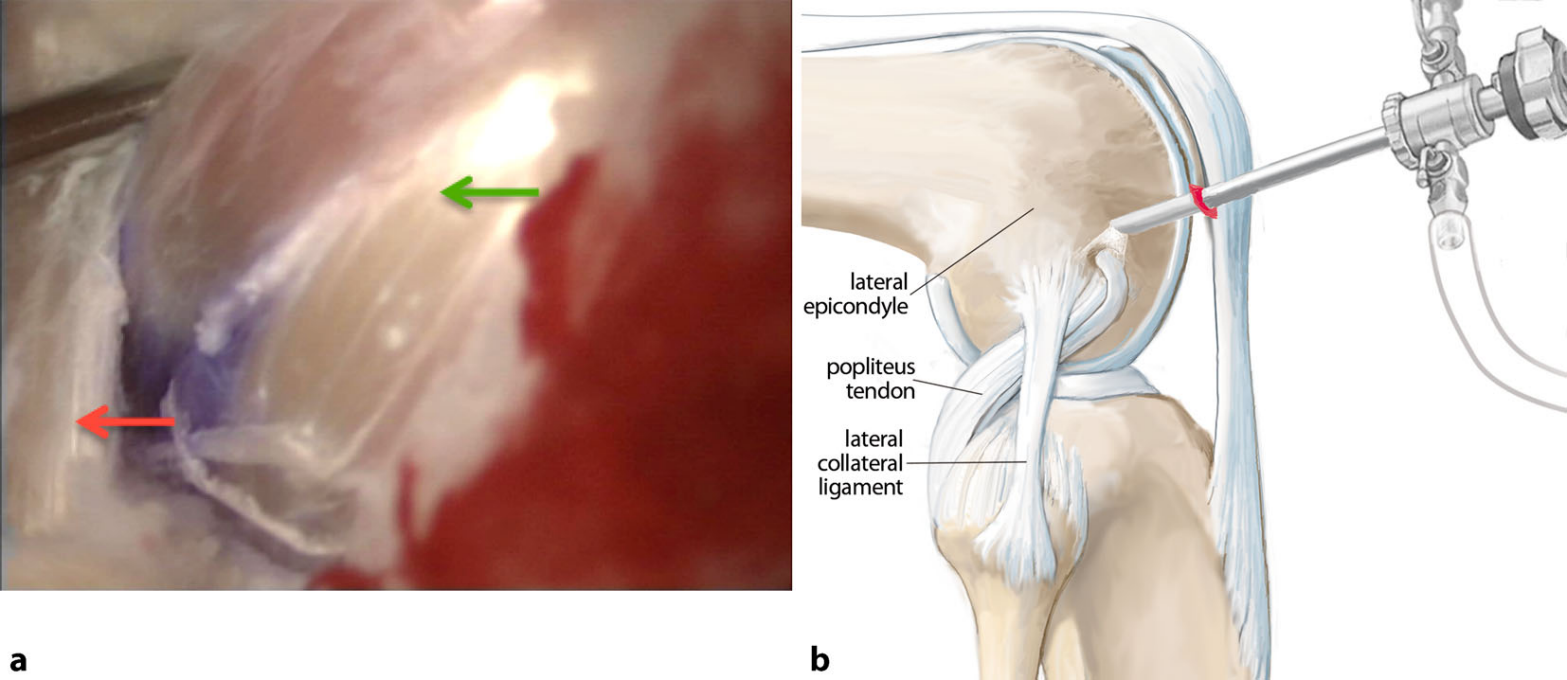

**Figure 15:**
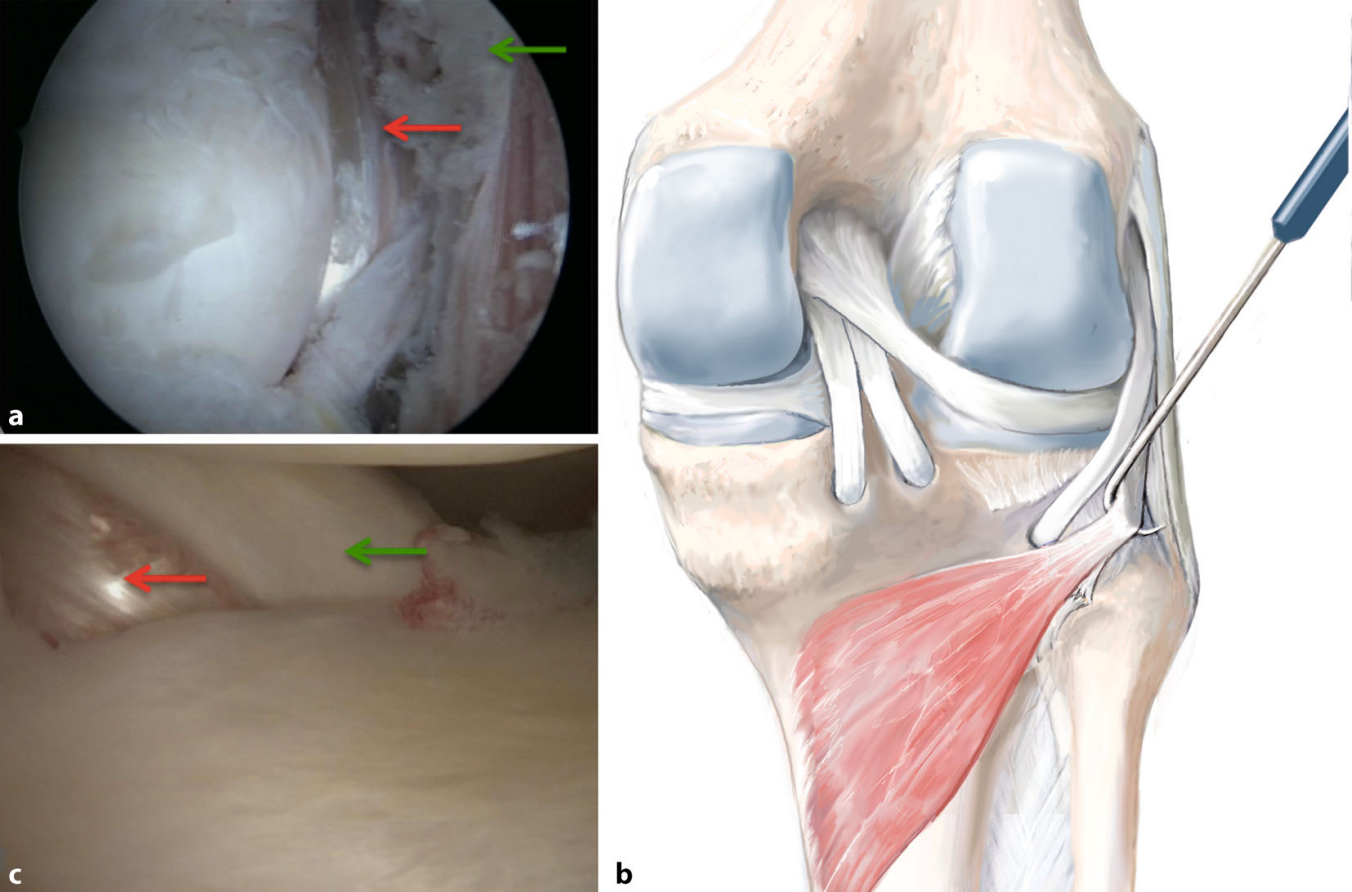

**Figure 16:**
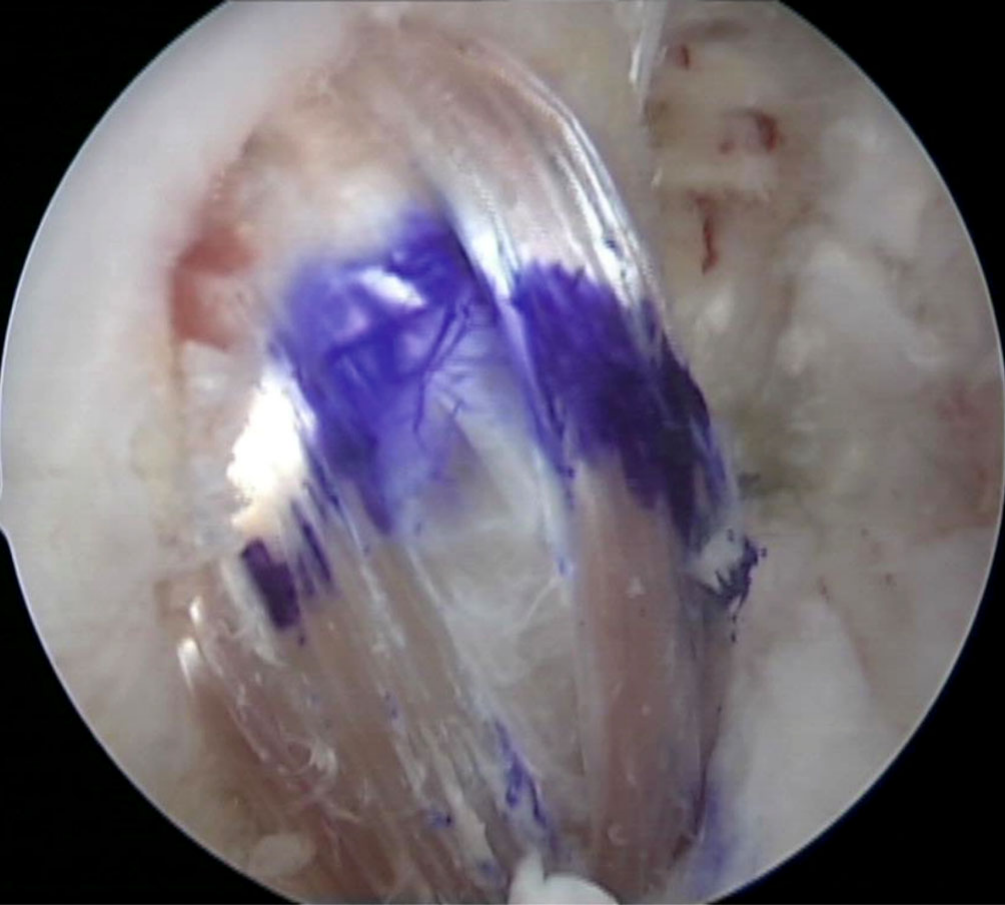

## Postoperative management

- Wound dressing until postoperative day 2
- Partial weight-bearing (10–20 kg) for 6 weeks
- PCL brace for 3 months (i. e., Jack PCL, Albrecht, Unterschleißheim, Germany) with limited range of motion 0–0–20° for 2 weeks, 0–0–45° for 2 weeks and 0–0–60° for further 2 weeks. 0–0–90° until week 8 and then free range of motion.
- Range-of-motion exercises in the prone position and passive flexion against quadriceps contraction up to 60° allowed from postoperative day 1
- Quadriceps strengthening exercises are allowed from postoperative day 1
- Active knee flexion is not allowed for the first 6 weeks postoperatively
- Proprioception loading exercises should be included
- Running and squatting exercises are begun after 3 months from the index procedure

## Results

To date, 35 patients have received a popliteus bypass graft due to a posterolateral rotational instability in combination with a PCL reconstruction. No intra- or postoperative complications have been observed so far. After 1 year, 12 patients (6 women) were examined (study still continuing). The mean age was 35.3 (± 13.6) years with a mean body mass index of 27.1 (± 3.6). The mean time from trauma to surgery was 11 (3–42) weeks. Among all patients who underwent surgery as described above, 3 patients received an additional LCL reconstruction, 1 patient underwent an additional ACL reconstruction, 1 patient had an additional high tibial osteotomy due to 7° of varus deformity (one-stage procedure), and 1 patient had an additional torsional osteotomy of the femur due to torsional deformity after femoral shaft fracture (two-stage procedure). The mean postoperative Lysholm Score was 88.4 (± 8.7) points, whereas the mean Tegner Score was preoperatively 5.6 (± 1.8) and 4.9 (± 1.0) points during follow-up. The Visual Analog Scale function was 2.8 (± 1.5; 0 complete function, 10 no function) and the Visual Analog Scale pain was 1.9 (± 1.8; 0 no pain, 10 maximal pain). In the preoperative stress x-rays with the Telos device, the mean side-to-side difference in the posterior drawer test in 90° of flexion was −13.3 (± 1.9) mm and postoperatively the mean side-to-side difference was −2.9 (± 2.2) mm. The Dial Test was negative in 10 of 12 patients.

The arthroscopic technique of posterolateral corner reconstructions has a low complication rate and leads to good and excellent clinical results.
